# Inducible gene inactivation in neurons of the adult mouse forebrain

**DOI:** 10.1186/1471-2202-8-63

**Published:** 2007-08-02

**Authors:** Gitta Erdmann, Günther Schütz, Stefan Berger

**Affiliations:** 1 Division Molecular Biology of the Cell I, German Cancer Research Center (DKFZ), Im Neuenheimer Feld 280, 69120 Heidelberg, Germany

## Abstract

**Background:**

The analysis of the role of genes in important brain functions like learning, memory and synaptic plasticity requires gene inactivation at the adult stage to exclude developmental effects, adaptive changes or even lethality. In order to achieve temporally controlled somatic mutagenesis, the Cre/loxP-recombination system has been complemented with the tamoxifen-inducible fusion protein consisting of Cre recombinase and the mutated ligand binding domain of the human estrogen receptor (CreER^T2^). To induce recombination of conditional alleles in neurons of the adult forebrain, we generated a bacterial artificial chromosome-derived transgene expressing the CreER^T2 ^fusion protein under control of the regulatory elements of the CaMKIIα gene (CaMKCreER^T2 ^transgene).

**Results:**

We established three mouse lines harboring one, two and four copies of the CaMKCreER^T2 ^transgene. The CaMKCreER^T2 ^transgene displayed reliable and copy number-dependent expression of Cre recombinase specifically in neurons of the adult forebrain. Using Cre reporter mice we show very low background activity of the transgene in absence of the ligand and efficient induction of recombination upon tamoxifen treatment in all three lines. In addition, we demonstrate in mice harboring two conditional glucocorticoid receptor (GR) alleles and the CaMKCreER^T2 ^transgene spatially restricted loss of GR protein expression in neurons of the adult forebrain upon tamoxifen treatment.

**Conclusion:**

This is to our knowledge the first approach allowing highly efficient inducible gene inactivation in neurons of the adult mouse forebrain. This new approach will be a useful tool to dissect the function of specific genes in the adult forebrain. Effects of gene inactivation on pre- and postnatal brain development and compensatory mechanisms elicited by an early onset of gene inactivation can now be excluded.

## Background

The generation of mouse mutants harboring targeted inactivation of desired genes using homologous recombination in embryonic stem cells is a powerful tool to analyze their role in complex brain functions such as learning and memory, synaptic plasticity as well as neurogenesis and neuronal cell death [1-3]. However, inactivation of genes in the germline often results in a lethal phenotype that prevents further analysis of the targeted gene in the adult brain.

To bypass early lethality and to analyze functions of a gene particularly in the adult brain the Cre/loxP-recombination system, that allows to conditionally ablate a defined gene, was implemented. In the Cre/loxP-recombination system the bacteriophage P1 Cre recombinase is expressed in a cell-type specific manner. In the Cre expressing cells the recombinase mediates excision of an essential part of the targeted gene that has been flanked by two loxP recognition sequences in the same orientation [4,5].

To drive the expression of Cre in the brain, in most cases short promotor fragments of genes with desired expression pattern have been cloned in front of a Cre cassette in a plasmid [6-8]. Since plasmid-derived transgenes show copy number-independent and often mosaic and ectopic expression [9,10] the use of BAC (bacterial artificial chromosome)- or YAC (yeast artificial chromosome)-derived transgenes is in favor. In contrast to plasmids, BAC and YAC vectors harbor large genomic regions containing almost all regulatory elements of the gene locus that was chosen to drive Cre expression. However, a given gene locus may not only be expressed in the adult brain, but also during pre- and postnatal development [11]. To overcome the necessity to find a gene locus that is active only at a desired time-point during development or at the adult stage fusion proteins consisting of Cre recombinase and a mutated ligand-binding domain (LBD) of a steroid hormone receptor have been developed to achieve ligand-dependent Cre activity. The mutated LBD retains the fusion protein in the cytoplasm and upon binding of a synthetic ligand it translocates into the nucleus, where it mediates the excision of the loxP-flanked DNA sequence [12-15]. It has been shown *in vitro *and *in vivo *that the CreER^T2 ^is the most potent Cre fusion protein with low leakiness and highly efficient induction [13,16]. The CreER^T2 ^fusion protein was so far applied in peripheral cell-types/tissues [17-19] and within the nervous system in oligodendrocytes and Schwann cells [20], astrocytes [21-23] and neural stem cells [24]. The verification of CreER^T2^-mediated recombination was only performed using Cre reporter mice. Cre reporter data are very helpful to detect ligand-independent (background) activity of the Cre and robust induction of recombination, but this type of analysis does not allow to assess incomplete induction. To analyze the extent of gene inactivation, expression analysis of the protein encoded by the targeted gene is mandatory.

In order to target neurons in the adult brain that participate in important brain functions like learning, memory and long term potentiation, we have chosen the CaMKIIα gene regulatory elements to drive CreER^T2 ^expression in the forebrain including limbic structures like hippocampus and amygdala. We introduced the CreER^T2 ^fusion protein in frame at the ATG of the CaMKIIα gene present on a large genomic fragment in a BAC vector using homologous recombination in bacteria. The established transgenic mouse lines (CaMKCreER^T2^) displayed copy number-dependent, forebrain-specific expression of the Cre recombinase. Using this transgene we show efficient forebrain-specific induction of recombination upon tamoxifen treatment with very low background activity in absence of the ligand restricted to the hippocampus. Unlike previous studies utilizing CreER^T2 ^mediated recombination in the brain, we demonstrate for the first time by expression analysis of the protein encoded by the targeted gene, the high efficiency of this inducible gene inactivation system.

## Results

### Copy number-dependent expression of the CreER^T2^-fusion protein in the adult mouse hippocampus

**Figure 1:**
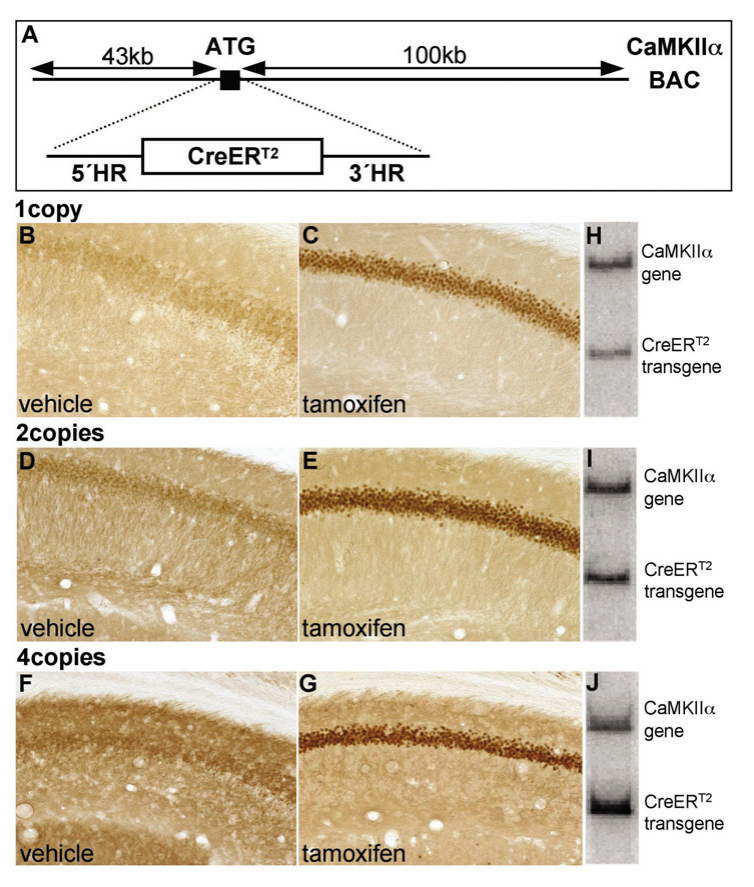
**Copy number-dependent transgene expression and tamoxifen-dependent nuclear translocation of the CreER^T2^-fusion protein in neurons of the adult hippocampus**. (A) The cassette encoding the CreER^T2^-fusion protein was inserted in frame at the ATG of the CaMKIIα gene present on a large DNA fragment of a BAC vector containing mouse genomic DNA by homologous recombination in bacteria. The genomic insert of the BAC vector contained a 43 kb 5'-upstream and a 100 kb 3'-downstream region of the CaMKIIα gene. (B-G) Immunohistochemical staining of pyramidal neurons in the hippocampal CA1 region of CaMKCreER^T2 ^transgenic animals with different transgene copy numbers using an antibody against Cre recombinase revealed non-nuclear localization of the Cre recombinase in vehicle-injected animals (B, D, F) and nuclear localization in animals 12 hours after injection of 1 mg tamoxifen (C, E, G). The CaMKCreER^T2 ^transgene copy number was determined by Southern blot analysis using a probe that detects restriction fragments of different sizes for the endogenous CaMKIIα gene (upper band) and the CaMKCreER^T2 ^transgene (lower band) (H, I, J).

To achieve inducible recombination of conditional alleles in neurons of the adult brain, we generated a BAC-derived transgene expressing the CreER^T2 ^fusion protein under the control of the regulatory elements of the mouse CaMKIIα gene (CaMKCreER^T2^). The CreER^T2 ^fusion protein consists of the Cre recombinase and a mutated ligand binding domain (LBD) of the human estrogen receptor (ER) [13,16]. The ER-LBD contains a nuclear localization signal that is unmasked upon ligand binding. Two point mutations in the ER-LBD allow binding of the synthetic ligand tamoxifen and prevent activation of the CreER^T2 ^by endogenous estradiol. Therefore, the unliganded form of the CreER^T2 ^fusion protein resides in the cytoplasm and upon tamoxifen binding it translocates into the nucleus and mediates site-specific recombination. We selected a BAC vector harboring a genomic insert containing the CaMKIIα gene locus with a 43 kb 5'upstream and a 100 kb 3'downstream region from the mouse genome project [25]. To obtain the CaMKCreER^T2 ^transgene we inserted the coding sequence for the CreER^T2 ^fusion protein at the ATG of the CaMKIIα locus (Fig. 1A) by homologous recombination in bacteria [26]. Using an equivalent genomic fragment containing the CaMKIIα gene our group has already generated a constitutive transgene (CaMKCre) that expresses the Cre recombinase under the control of the regulatory elements of the mouse CaMKIIα gene [27].

The modified genomic insert was released and separated from the BAC backbone and then introduced into the mouse germline by oocyte injection. Transgenic founder animals were identified by PCR and dot blot analysis of tail DNA. The F1 progeny was used to determine the copy number of the transgene by Southern blot (Fig. 1H–J). Three lines harboring either one, two or four copies of the transgene were chosen for further analyses. We investigated the expression and nuclear translocation of the CreER^T2 ^fusion protein upon tamoxifen treatment by immunohistochemistry on hippocampal vibratome sections using an anti-Cre antibody. Transgenic animals were injected with either tamoxifen or vehicle 12 hours before dispatch. In the vehicle-treated animals the CreER^T2 ^protein was located in the cytoplasm (Fig. 1B, D and 1F), whereas tamoxifen-treated animals displayed nuclear localization of the Cre recombinase in the hippocampus (Fig. 1C, E and 1G). In addition, copy number-dependent expression was observed, the four-copy line showed stronger expression of the CreER^T2 ^fusion protein than the two-copy line which displayed a stronger Cre expression in the hippocampus than the one-copy line (Fig. 1B–J). Nuclear translocation and copy number-dependent CreER^T2 ^expression was also observed in other brain regions, e.g. cortex (data not shown).

### Targeted recombination in adult forebrain regions upon tamoxifen induction

To assess the ER^T2^-mediated control of Cre activity in absence of the ligand in forebrain neurons and to examine the efficiency of tamoxifen-induced Cre-mediated recombination, CaMKCreER^T2 ^mice with different transgene copy numbers were crossed with ROSA26 Cre reporter mice (R26R allele) [28] to obtain R26R/CaMKCreER^T2 ^mice. The Cre reporter line harbors a knock-in of a lacZ gene preceded by a stop cassette, flanked by two recognition sequences (loxP) for the Cre recombinase, in the ROSA26 locus. Upon tamoxifen treatment CreER^T2 ^translocates into the nucleus and deletes the stop cassette thereby permitting β-galactosidase expression.

**Figure 2:**
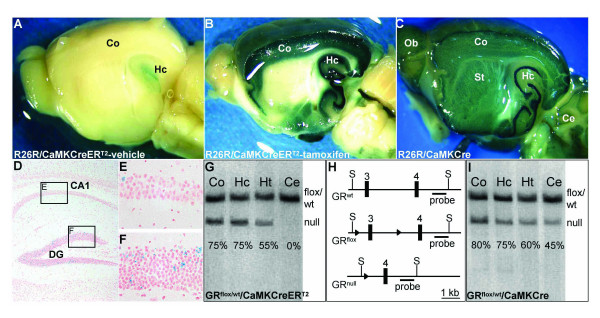
**CreER^T2 ^fusion protein expression driven by the regulatory elements of the CaMKIIα gene allows targeting of specific regions in the adult brain upon tamoxifen treatment**. (A, B) β-galactosidase staining of brain slices revealed that animals harboring a ROSA26 Cre reporter allele and the CaMKCreER^T2 ^transgene (R26R/CaMKCreER^T2^, two copies) show low Cre activity in absence of the ligand only within the hippocampus (Hc) (A), but strong staining within the cortex (Co) and hippocampus (Hc) upon tamoxifen treatment (B). (D-F) Analysis of β-galactosidase positive neurons using cryosections of the hippocampus of R26R/CaMKCreER^T2 ^mice revealed sparse recombination in the granular neurons of the dentate gyrus (DG) and in hippocampal neurons of the CA1 region in the absence of tamoxifen. (C) β-galactosidase staining of a brain slice isolated from an animal harboring a ROSA26 Cre reporter allele and the constitutive CaMKCre transgene (R26R/CaMKCre) revealed recombination in the hippocampus and cortex as well as recombination in olfactory bulbs (Ob), striatum (St) and cerebellum (Ce). (G-I) Recombination of a conditional GR allele achieved using the CaMKCreER^T2 ^transgene (two copies) plus tamoxifen treatment (2 × 1 mg tamoxifen for five consecutive days) is comparable to recombination obtained using the constitutive CaMKCre transgene. Recombination levels detected in distinct brain regions (Co, Hc, Ht = hypothalamus, Ce) of mice heterozygous for the conditional GR allele (GR^flox^) and either the constitutive (GR^flox/wt^/CaMKCre) (I) or the inducible (GR^flox/wt^/CaMKCreER^T2^, tamoxifen-treated) (G) Cre transgene (two copies) were determined using Southern blot analysis. The Southern blot strategy is depicted in (H), black boxes represent exons three and four of the GR gene, the arrowheads the loxP sites and S marks SacI recognition sites. The probe detects in case of the GR^flox ^and the GR^wt ^allele a DNA fragment of similar size and in case of the GR^null ^allele a smaller DNA fragment. The bands were quantified by phosphoimager and the percentage of recombination for each region is indicated. Animals for Cre reporter and Southern blot analysis were treated with 1 mg tamoxifen or vehicle twice a day for five consecutive days and sacrificed ten days after the last injection.

R26R/CaMKCreER^T2 ^mice were injected twice per day with either tamoxifen or vehicle for five consecutive days. Whole mount samples of the adult brain from the one-, two- or four-copy line were analyzed for β-galactosidase activity. Determination of CreER^T2 ^activity in absence of the ligand revealed that ten weeks old vehicle-injected animals of all three transgenic lines showed very little β-galactosidase activity within the hippocampus only. Using whole mount staining we could not detect substantial differences between one, two and four copies of the transgene (Fig. 2A, two-copy line). After tamoxifen treatment in all three lines β-galactosidase activity was most prominent in the cortex and hippocampus. The one-copy line showed less intense staining than the two- and four-copy lines. However, no apparent difference was observed between the two-copy and the four-copy line (data not shown). In all three lines no β-galactosidase activity was detected in other organs than the brain after tamoxifen induction (data not shown).

Since the use of the minimal number of transgene copies that allow efficient recombination upon tamoxifen induction is desired, we only characterized for the two-copy line the activity in absence of tamoxifen ("background") in more detail. At the cellular level, using cryosections, vehicle-treated R26R/CaMKCreER^T2 ^mice of the two-copy line displayed only few granular neurons in the dentate gyrus and even less pyramidal neurons in the CA1 region of the hippocampus that underwent recombination of the reporter allele (Fig. 2D–F).

In order to compare the spatial activity of the inducible CaMKCreER^T2 ^transgene with the constitutive CaMKCre transgene, β-galactosidase stained brain whole mount samples of adult R26R/CaMKCre mice were analyzed as well. Both, R26R/CaMKCreER^T2 ^(two copies) and R26R/CaMKCre mice showed extensive recombination in hippocampus and cortex (Fig. 2B and 2C). However, the constitutive transgene caused additional recombination in olfactory bulbs, striatum and cerebellum (Fig. 2C). Immunohistochemical staining for Cre revealed a strong expression of CreER^T2 ^protein in those regions showing recombination in R26R/CaMKCreER^T2 ^mice (e.g. hippocampus, cortex). As shown for the constitutive CaMKCre line at the adult stage [27], we could detect only very low level of CreER^T2 ^expression in striatum and thalamus and no expression in cerebellum of CaMKCreER^T2 ^transgenic mice (data not shown). Using Cre reporter mice we did not observe Cre-mediated recombination in any of these regions after tamoxifen application (data not shown).

To quantify and compare recombination evoked by the constitutive CaMKCre transgene and the CaMKCreER^T2 ^transgene, induced with 1 mg tamoxifen twice per day for five consecutive days, at the DNA level by Southern blot, we generated animals heterozygous for the conditional GR allele (GR^flox^) [29] harboring either the constitutive (GR^flox/wt^/CaMKCre) or the inducible Cre (GR^flox/wt^/CaMKCreER^T2^, two copies) transgene respectively. The analysis of adult samples of different brain regions revealed that the areas showing recombination using Cre reporter mice (e.g. hippocampus and cortex) display a comparable degree of recombination for both transgenes (Fig. 2G–I). In case of the cerebellum we detected, as expected from the Cre reporter data, no recombination of the GR^flox ^allele for the induced CaMKCreER^T2 ^transgene, but distinct recombination for the constitutive CaMKCre transgene (Fig. 2G and 2I).

### Ablation of GR protein expression in the adult brain upon tamoxifen treatment using the CaMKCreER^T2 ^transgene

**Figure 3:**
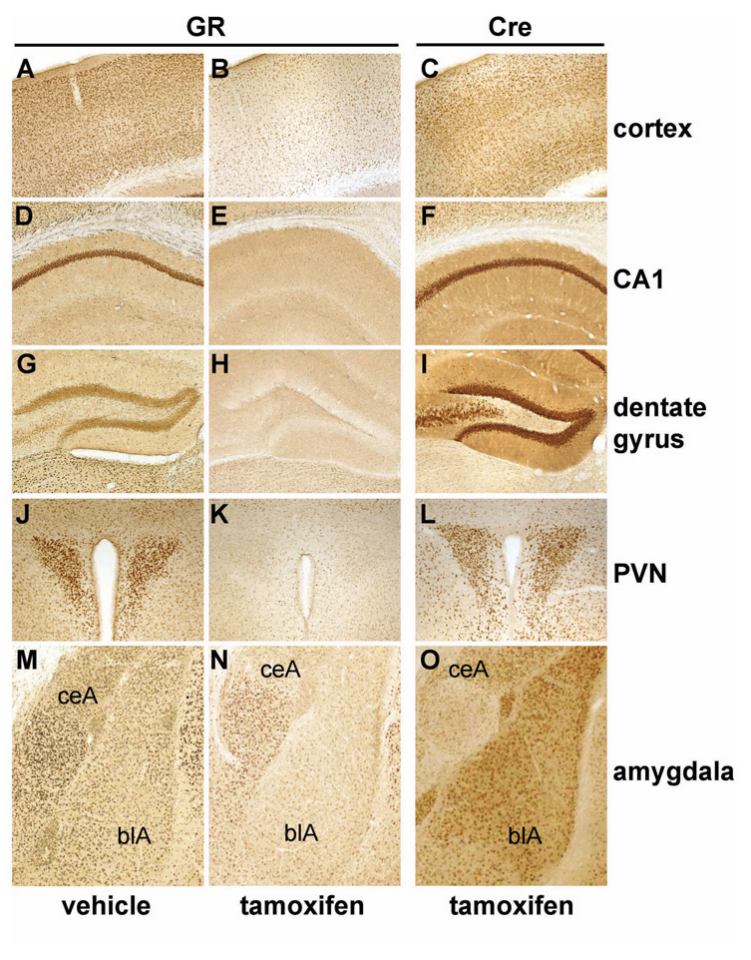
**Ablation of GR protein expression in neurons of the adult brain upon tamoxifen treatment**. Vibratome-sections of mice homozygous for a conditional GR allele (GR^flox^) and heterozygous for the CaMKCreER^T2 ^transgene (GR^CaMKCreERT2 ^mice) have been analyzed by immunohistochemistry. GR protein is expressed in vehicle-treated GR^CaMKCreERT2 ^mice (A, D, G, J, M), whereas loss of GR expression upon tamoxifen treatment is observed in brain regions (B, E, H, K, N) that display strong CreER^T2 ^expression (C, F, I, L, O). The analyzed GR^CaMKCreERT2 ^mice harbor two copies of the CaMKCreER^T2 ^transgene. Depicted are brain regions that have been identified as target regions using ROSA26 reporter mice and/or show strong GR expression in controls: cortex (A-C), CA1 region of the hippocampus (D-F), dentate gyrus (G-I), PVN (J-L) as well as central (ceA) and basolateral amygdala (blA) (M-O). The animals were injected with 1 mg tamoxifen or vehicle twice a day for five consecutive days and sacrificed ten days after the last injection. In addition, Cre transgenic mice were injected with tamoxifen 12 hours before analysis to obtain a nuclear staining for the CreER^T2 ^fusion protein.

To demonstrate at the cellular level that the tamoxifen-induced CreER^T2^-mediated recombination of a targeted gene results in complete protein loss within specific populations of neurons, we generated GR^flox/flox^/CaMKCreER^T2 ^(GR^CaMKCreERT2^) mice with two copies of the CaMKCreER^T2 ^transgene. Eight weeks old GR^CaMKCreERT2 ^mice were treated twice per day either with 1 mg tamoxifen or vehicle for five consecutive days and were sacrificed ten days after tamoxifen application. In addition, the mice were injected with 1 mg tamoxifen 12 hours before dispatch to visualize Cre expression. Immunohistochemistry revealed CreER^T2 ^expression in the cortex, the hippocampus (Fig. 3C, F and 3I) as well as in the hypothalamic paraventricular nucleus (PVN) (Fig. 3L) and basolateral amygdala, whereas the central amygdala was spared (Fig. 3O). Accordingly, in neurons of these regions with strong CreER^T2 ^expression, tamoxifen-treated animals show loss of GR protein (Fig. 3B, E, H, K and 3N) compared to vehicle-treated mice (Fig. 3A, D, G, J and 3M). Analysis of CreER^T2 ^and GR protein expression in other forebrain structures revealed strong CreER^T2 ^expression and the corresponding GR protein loss in the lateral septum and in the medial preoptic area, whereas in the striatum only weak Cre expression and no visible loss of GR protein were detectable (data not shown). Even though Cre reporter data revealed CreER^T2 ^activity in absence of the ligand in the hippocampus, the background activity is much too low to allow the detection of a visible loss of GR protein expression in vehicle-treated GR^CaMKCreERT2 ^mice in comparison to wild-type animals (data not shown).

To identify neuronal subtypes that are targeted in the cortex by the CaMKCreER^T2 ^transgene, we performed immunohistochemical double-stainings for CreER^T2 ^and markers for the two major neuronal subtypes present in the cortex. GABAergic interneurons were identified by GAD65/67 expression and glutamatergic neurons by VGLUT1 expression. We detected no CreER^T2 ^protein expression in GABAergic interneurons, whereas expression of CreER^T2 ^was present in glutamatergic neurons (data not shown).

### Effect of variations in transgene copy number and the applied tamoxifen dose on GR protein loss

It has been shown previously that efficient induction of recombination is dependent on the applied tamoxifen dose and the level of CreER^T2^expression as well as on the particular conditional allele [16,20,30]. Thus it is possible, that different conditional alleles require different levels of CreER^T2 ^activity. The level of CreER^T2 ^activity is dependent on the expression level of the CreER^T2 ^protein and on the availability of tamoxifen. Since the CaMKCreER^T2 ^transgene shows copy number-dependent CreER^T2 ^expression, we determined if we can detect an interrelation of transgene copy number and tamoxifen dose for the recombination evoked by this transgene. For this purpose, we injected GR^CaMKCreERT2 ^mice harboring one, two or four copies of the transgene with 1 mg tamoxifen for five consecutive days once per day (5 mg) or twice per day (10 mg) and analyzed the loss of GR protein in hippocampus, cortex and PVN.

**Figure 4:**
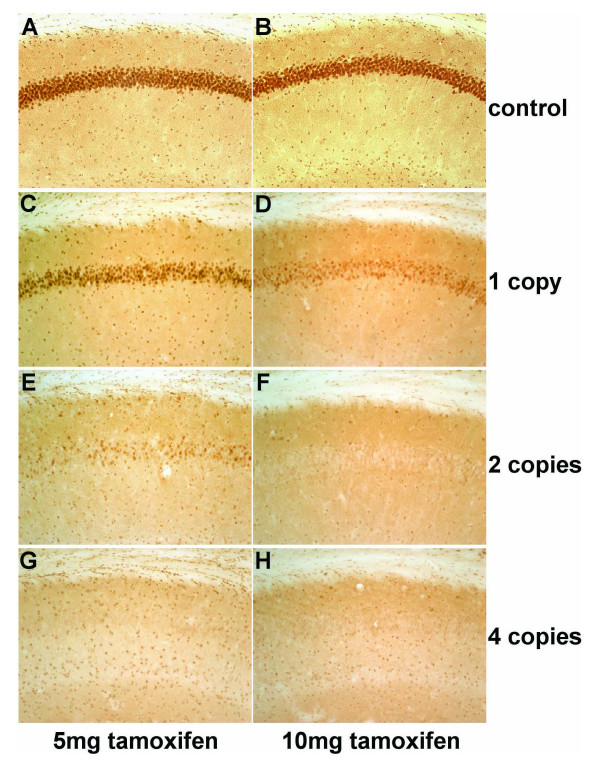
**CreER^T2^-mediated recombination within the adult hippocampus depends on the transgene copy number and the tamoxifen dose**. Pyramidal neurons in the hippocampal CA1 region of GR^CaMKCreERT2 ^and control animals were analyzed by immunohistochemistry on vibratome-sections using an antibody against GR. Mice were injected with tamoxifen for five consecutive days either once per day (5 mg) (A, C, E, G) or twice per day (10 mg) (B, D, F, H) and sacrificed ten days after the last injection. Treatment with 5 mg tamoxifen results in an incomplete loss of GR protein if one or two copies of the CaMKCreER^T2 ^transgene are present (C, E), whereas four copies of the transgene are sufficient to induce complete GR protein loss (G). GR^CaMKCreERT2 ^mice harboring two or four copies of the Cre transgene display complete loss of GR protein after injection of 10 mg tamoxifen (F, H). A visible but still incomplete loss of GR protein was seen in CaMKCreER^T2 ^transgenic mice of the one-copy line treated with 10 mg tamoxifen (D) whereas tamoxifen-treated controls show no apparent loss of GR (A, B).

The immunohistochemical analysis of the pyramidal neurons of the hippocampal CA1 region revealed that tamoxifen-treated controls (GR^flox/flox^, Fig. 4A and 4B) show no difference in GR protein expression compared to wild-type mice (data not shown). GR^CaMKCreERT2 ^mice with one copy of the transgene (Fig. 4C) show moderate loss of GR signal after applying 5 mg tamoxifen, but a strong decrease of GR protein if 10 mg tamoxifen were injected (Fig. 4D). Mutant mice with two CaMKCreER^T2 ^copies treated with 5 mg tamoxifen also show a strong but incomplete loss of GR protein (Fig. 4E), comparable to one-copy animals treated with 10 mg tamoxifen (Fig. 4D). GR^CaMKCreERT2 ^animals with two and four copies of the transgene display a complete loss of GR after injection of 10 mg tamoxifen (Fig. 4F and 4H). Likewise, GR^CaMKCreERT2 ^mice with four copies of the transgene treated with 5 mg tamoxifen show complete GR loss in pyramidal neurons of the hippocampal CA1 region (Fig. 4G). The same correlation between copy number and ligand dose was observed in the dentate gyrus of the hippocampus and in the cortex (data not shown). However, in the PVN complete loss of GR signal was detected upon injection of 10 mg tamoxifen in the presence of two or four copies only, but none of the lines showed complete GR loss if 5 mg were applied (data not shown). Taken together, these data demonstrate that increasing copies of the transgene reduce the tamoxifen dose required to achieve recombination.

It has been shown previously, that ablation of GR protein in neurons and glia cells results in a strong elevation of plasma corticosterone [29]. The adrenal release of corticosterone is controlled by the hypothalamic-pituitary-adrenal (HPA) axis. The feedback regulation of the HPA axis is mediated by GR at the level of the pituitary and PVN [31]. Since the PVN can be targeted with the CaMKCreER^T2 ^transgene, the measurement of plasma corticosterone provides information about the efficiency of the GR ablation using the CaMKCreER^T2 ^transgene. Therefore, we measured plasma corticosterone of GR^CaMKCreERT2 ^animals harboring one, two or four copies of the transgene and their littermate controls (GR^flox/flox^) at the circadian trough. All animals were injected with 1 mg tamoxifen twice a day for five consecutive days and sacrificed six weeks after the treatment. The mutant mice from all three lines show, as expected, a significant elevation of plasma corticosterone compared to controls (Table 1). Mutant mice, harboring one copy of the transgene display a significant, but rather mild elevation of corticosterone. GR^CaMKCreERT2 ^mice with two and four copies of the transgene exhibit an equivalent and compared to mutant animals with one copy of the transgene a stronger elevation of the morning corticosterone level (Table 1).

**Table 1:** Plasma corticosterone level [ng/ml] of adult tamoxifen-treated GR^CaMKCreERT2 ^mice at diurnal trough

**line**	**control**	**mutant**	**vs. one copy**	**vs. two copies**
one copy	13 ± 5	39 ± 8*		
	n = 7	n = 9		
two copies	8 ± 1	100 ± 20*	p < 0.05	
	n = 7	n = 9		
four copies	15 ± 6	108 ± 19*	p < 0.05	p = 0.75
	n = 5	n = 5		

control = GR^flox/flox^, mutant = GR^flox/flox^/CaMKCreER^T2 ^transgene

* p < 0.05, control versus mutant

## Discussion

Brain-specific gene inactivation using the Cre/loxP-recombination system allows to investigate gene function in the adult brain in case the germline inactivation of the gene of interest causes lethality. However, most of the available brain-specific Cre-lines show onset of Cre expression before the development of the brain is completed [6,29,32]. Therefore, phenotypical changes can result from interferences during brain development and/or from deficiency of the respective protein in the adult brain [11].

To achieve a spatio-temporal control of Cre-mediated recombination in the adult brain, regulatory elements of the CaMKIIα gene have been already used to drive the expression of different Cre fusion proteins. The fusion of Cre recombinase with a modified ligand-binding domain (LBD) of a steroid hormone receptor (e.g. progesterone receptor or estrogen receptor) allows to regulate nuclear translocation and thereby activity of the Cre recombinase by administration of the cognate ligand. Expression of Cre recombinase fused to a mutated form of the human progesterone receptor-LBD resulted in incomplete recombination within the hippocampal cornu ammonis and the cortex upon RU 486 treatment, but no recombination was observed in the dentate gyrus [14]. The fusion of a mutated ligand-binding domain of the estrogen receptor (ER^T2^) [13] onto both ends of the Cre recombinase, expressed under the control of the regulatory elements of the CaMKIIα gene, resulted in very weak recombination efficiency (5%–10% in hippocampal pyramidal and granular neurons) upon tamoxifen treatment [33]. The fusion of one ER^T2 ^to the C-terminal end of Cre recombinase [13] has been recently employed in oligodendrocytes, astrocytes and neural stem cells [20-24]. We have now generated transgenic mouse lines expressing the CreER^T2^fusion protein under the control of regulatory elements of the mouse CaMKIIα gene present on a large genomic DNA fragment isolated from a BAC library. This transgene allows highly efficient recombination of conditional genes in brain regions shown to be important for learning, memory and synaptic plasticity.

### The CaMKCreER^T2 ^transgene displays forebrain-specific, copy number-dependent expression and permits inducible site-specific recombination

The use of plasmid-derived transgenes that contain small promoter fragments in front of an expression cassette results in copy number-independent transgene expression. In addition, variable degrees of ectopic and mosaic expression of the transgene is observed [9,10,34]. The use of BAC or YAC clones, that contain large regions of genomic DNA with almost all regulatory elements of the gene with desired expression in their native configuration, allows expression of the transgene that resembles the expression of the endogenous gene independent of the integration site [27,35,36]. Using the regulatory elements of the mouse CaMKIIα gene we have generated transgenic mice expressing the CreER^T2 ^fusion protein in neurons of the adult forebrain. We obtained different lines harboring one, two or four copies of the transgene. As expected, the expression pattern of the transgene is identical in all analyzed transgenic lines and shows only copy number-dependent differences at the level of expression.

Using the ROSA26 Cre reporter we demonstrated the effective temporal regulation of CreER^T2 ^activity by tamoxifen treatment in all three lines. In absence of the ligand noteworthy constitutive recombination occurs only within the dentate gyrus. Application of tamoxifen results in strong recombination within forebrain regions like hippocampus and cortex. Using the two-copy line we compared the degree of recombination with the one elicited by the constitutive CaMKCre line. Analysis of β-galactosidase stainings of double transgenic animals harboring a ROSA26 Cre reporter allele and the constitutively active CaMKCre revealed recombination in the entire brain. However, brain regions that show recombination in both, the constitutive and the inducible Cre transgene after tamoxifen treatment, display in the adult brain (ten weeks) a comparable degree of recombination when quantified by Southern blot. Since both transgenic lines show similar Cre expression, independent of the developmental stage, the observed differences in recombination pattern result from the different time period of nuclear Cre activity of both transgenes. Using a ROSA26 Cre reporter, we observe in case of the constitutive CaMKCre transgene the accumulated recombination and thereby β-galactosidase staining in all cells where Cre was active during development and adulthood until dispatch. Therefore, brain regions that show no or only weak Cre expression at the adult stage can nevertheless show β-galactosidase staining. In case of the CaMKCreER^T2 ^transgene Cre is only transiently activated during the presence of tamoxifen. Thus the pattern of recombination reflects CreER^T2 ^expression within this time period only.

### Inducible ablation of GR protein expression in the adult brain depends on the copy number of the Cre transgene and the tamoxifen dose

We successfully employed the CaMKCreER^T2 ^transgene in combination with a conditional GR allele (GR^CaMKCreERT2 ^mice, two copies of the CreER^T2 ^transgene) to achieve ablation of GR in neurons of the adult forebrain. Upon tamoxifen treatment, GR^CaMKCreERT2 ^mice show loss of GR protein, whereas vehicle-treated mutants showed no alterations in GR expression compared to wild-type mice, reflecting the very low background activity of the CaMKCreER^T2 ^transgene. In addition, we demonstrate for the hippocampus that an increase in transgene copy numbers allows the reduction of the tamoxifen dose required to achieve complete recombination.

Using the conditional GR allele we could show that using the two- or four-copy line of the CaMKCreER^T2 ^transgene in combination with the injection of 1 mg tamoxifen twice per day for five consecutive days results in similar recombination efficiency, leading to complete loss of GR protein in the cortex, hippocampus, PVN and basolateral amygdala as well as a comparable increase of plasma corticosterone. Using the same tamoxifen dose the one-copy line displays only partial loss of GR protein in PVN and hippocampus resulting in a milder increase of plasma corticosterone. Since BAC-derived large genomic DNA fragments potentially carry additional genes we will use for further analyses of GR^CaMKCreERT2 ^mice two copies of the CaMKCreER^T2 ^transgene and apply 1 mg tamoxifen twice per day for five consecutive days. Moreover, we could induce MR protein loss upon the identical tamoxifen treatment in the brain regions targeted by the CaMKCreER^T2 ^transgene using the two-copy line in combination with a conditional MR allele [11] (unpublished observation). However, other conditional alleles may require the use of four copies of the CaMKCreER^T2 ^transgene in combination with the injection of 1 mg tamoxifen twice per day for five consecutive days, to achieve complete recombination.

## Conclusion

The expression of the CreER^T2^-fusion protein under control of the regulatory elements of the CaMKIIα gene (CaMKCreER^T2 ^transgene) allows inducible gene inactivation in neurons of the adult forebrain with very low background activity and highly efficient targeting upon tamoxifen treatment. The use of a BAC-based transgene resulted in a reliable and copy number-dependent transgene expression. By employing the CaMKCreER^T2 ^transgene in combination with a conditional GR allele we were able to induce spatially and temporally restricted loss of GR protein dependent on transgene copy number and applied tamoxifen dose.

## Methods

### Generation of CaMKCreER^T2 ^transgenic mice

A bacterial artificial chromosome (BAC) harboring 143 kb of genomic DNA (RP24-243J21) was chosen from the mouse genome project [25]. The genomic DNA-fragment contains, similar to the one used to generate a constitutive CaMKCre transgene [27], the mouse CaMKIIα locus with a 43 kb 5'upstream and a 100 kb 3'downstream region. This BAC was modified by homologous recombination to insert a cassette encoding a fusion protein (CreER^T2^) consisting of a codon-improved Cre recombinase (Cre) [37] and a mutated ligand-binding domain of the human estrogen receptor (ER^T2^) [13] as well as an ampicillin resistance cassette flanked by two FRT sites. The linear construct was electroporated into heat-induced EL250 bacteria [26] harboring the BAC. Clones with the recombinant BAC were induced with L-arabinose to express Flp recombinase that results in deletion of the ampicillin resistance cassette [26]. The modified genomic fragment containing the CreER^T2 ^knock-in at the ATG of the CaMKIIα gene was separated from the BAC backbone by NotI digestion and subsequent preparative pulse-field gel electrophoresis. The DNA was purified from the gel slice and microinjected into the pronucleus of FVB/N mouse oocytes [38]. Transgenic offspring (founder) was identified by dot blot of tail DNA. A fragment of the coding sequence of Cre was labeled and used as probe. Founder mice were bred with C57Bl/6 mice to generate BAC-transgenic (CaMKCreER^T2^) mouse lines. Three different lines with one, two and four copies of the transgene were analyzed. The Cre-transgenic mice were bred with ROSA26 Cre reporter mice or with mice harboring a conditional GR allele (GR^flox^) [29] to generate the respective genotypes used for the experiments.

### Animal-treatment

Tamoxifen (Sigma) was dissolved in sunflower seed oil/ethanol (10:1) mixture at a final concentration of 10 mg/ml. Eight weeks old mice were injected intraperitoneally with 1 mg of tamoxifen either once or twice per day for five consecutive days. Control animals were injected with 100 μl of sunflower seed oil/ethanol mixture (vehicle). If not otherwise specified, control and mutant mice possessed the same genotype, but differed in the treatment (control: vehicle, mutant: tamoxifen). Experimental animals for immunohistochemistry and Cre reporter analysis were sacrificed ten days after the last injection. For corticosterone measurement GR^flox/flox ^(control) and GR^flox/flox^/CaMKCreER^T2 ^(mutant) mice were both treated with 1 mg tamoxifen twice a day for five consecutive days and were sacrificed six weeks after the last injection to allow recovery from the tamoxifen treatment.

### Corticosterone measurement

To determine plasma corticosterone, adult mice (14 weeks) were housed individually one week before the experiment (light on 9:00 – 21:00). Blood sampling was performed in the morning (starting at 10:00) by bleeding after decapitation, with the time from first handling to decapitation not exceeding 20 s, to determine plasma corticosterone at circadian trough. Plasma corticosterone was measured using a commercially available RIA kit (MP Biomedicals). Results are presented as mean ± SEM and were analyzed using unpaired, two-tailed Student's *t *test.

### Immunohistochemistry and β-galactosidase staining

For immunohistochemical analysis, the dissected brains were postfixed with 4% paraformaldehyde at 4°C for 72 hrs. Coronal sections with a thickness of 50 μm were performed using a vibratome (Microm). The floating sections were processed for immunohistochemistry using the VECTASTAIN ABC system (Vector Laboratories) and diaminobenzidine (Sigma) incubation. Following primary antibodies have been used: polyclonal anti-Cre (diluted 1:3000) [14] and polyclonal anti-GR (diluted 1:1000) (Santa Cruz Biotechnology).

For β-galactosidase whole mount stainings, dissected brains were cut with a razor blade in the appropriate planes, washed three times with washing buffer (1 × PBS, 5 mM EGTA, 2 mM MgCl_2_, 0.01% sodium-deoxycholat and 0.02% NP-40) and were subsequently stained with X-gal staining solution (5 mM EGTA, 2 mM MgCl_2_, 0.01% sodium-deoxycholat, 0.02% NP-40, 10 mM K_3 _[Fe(CN)_6_], 10 mM K_4 _[Fe(CN)_6_] and 0.5 mg/ml X-gal in 1 × PBS) at 37°C over night. After the staining slices were postfixed with 4% paraformaldehyde at 4°C over night and kept in 1 × PBS at 4°C for analysis. For β-galactosidase staining of cryosections, dissected brains were frozen in O.C.T. compound (Tissue Tek, Sakura Finetek) on ethanol/dry ice and 10 μm sagital cryosections were prepared. Sections were fixed 10 min in 4% paraformaldehyde and β-galactosidase staining was performed as described for whole mount samples. Afterwards, sections were counterstained with nuclear fast red (Sigma) and mounted with Eukitt (O. Kindler).

### Analysis of transgene copy numbers and quantification of recombination

Transgene copy number was determined by Southern blot of tail DNA digested with BamHI and hybridized with a DNA probe detecting sequences in the CaMKIIα promotor region. The probe was labeled by random-priming with α-^32^PdCTP. Relative band intensities were assessed by phosphoimager (Fuji) and the band originating from the endogenous CaMKIIα gene was calculated as two copies.

For analysis of Cre-mediated recombination, genomic DNA was isolated from different brain areas. The DNA was digested with SacI, blotted and hybridized with a probe detecting in case of the GR^flox ^and the GR^wt ^allele a fragment of similar size (7 kb) and in case of the GR^null ^allele a smaller fragment (4.8 kb). Signals were quantified by phosphoimager (Fuji) and percentage of recombination is calculated as signal intensity of the GR^null ^band divided by the sum of the signal intensities of the GR^flox^/GR^wt ^band and GR^null ^band.

## Authors' contributions

GE participated in the generation of the CaMKCreER^T2 ^transgenic lines and coordinated the study, carried out the histological and functional characterization of the lines and wrote the manuscript. GS contributed to the design of the study and to the draft of the manuscript. SB conceived the study, generated the CaMKCreER^T2 ^transgene and designed the manuscript. All authors read and approved the final manuscript.
